# Probiotic Incorporation in Edible Films and Coatings: Bioactive Solution for Functional Foods

**DOI:** 10.3390/ijms19010150

**Published:** 2018-01-04

**Authors:** Foteini Pavli, Chrysoula Tassou, George-John E. Nychas, Nikos Chorianopoulos

**Affiliations:** 1 Institute of Technology of Agricultural Products, Hellenic Agricultural Organization-DEMETER, Lycovrissi, 14123 Attica, Greece; photpavli@gmail.com (F.P.); ctassou@nagref.gr (C.T.); 2 Laboratory of Food Microbiology and Biotechnology, Department of Food Science and Human Nutrition, Agricultural University of Athens, Iera Odos 75, 11855 Athens, Greece; gjn@aua.gr

**Keywords:** probiotics, edible films, edible coatings, bioactive packaging

## Abstract

Nowadays, the consumption of food products containing probiotics, has increased worldwide due to concerns regarding healthy diet and wellbeing. This trend has received a lot of attention from the food industries, aiming to produce novel probiotic foods, and from researchers, to improve the existing methodologies for probiotic delivery or to develop and investigate new possible applications. In this sense, edible films and coatings are being studied as probiotic carriers with many applications. There is a wide variety of materials with film-forming ability, possessing different characteristics and subsequently affecting the final product. This manuscript aims to provide significant information regarding probiotics and active/bioactive packaging, to review applications of probiotic edible films and coatings, and to discuss certain limitations of their use as well as the current legislation and future trends.

## 1. Introduction

Diet has a major impact on general health and, consequently, is integrated within public health strategies set up to promote optimum health conditions throughout life by preventing chronic diseases such as gastrointestinal disorders, cardiovascular disease, cancer and osteoporosis. Amongst major food categories commonly consumed, probiotic foods have a significant effect on gut health. Such food products can serve as a great example to highlight the strong connection between food and health condition. The consumers’ demand for healthy food is continuously increasing, hence, stimulating innovation and new product development in the food industry worldwide. This has increased the attention of researchers to study in depth the specific characteristics of certain food products and their effects on human health.

As defined by Food and Agriculture Organization of the United Nations/World Health Organization (FAO/WHO) in 2002, probiotics are “live microorganisms which, when administered in adequate amounts, confer health benefit on the host” [1]. Members of the genera *Bifidobacterium* and *Lactobacillus* are the most frequently used probiotics, while members of the genera *Streptococcus* and *Enterococcus* contain some opportunistic pathogens [2,3]. Furthermore, some yeasts such as *Saccharomyces boulardii* are generally accepted for use as probiotics. Since the dairy sector has pioneered the introduction of probiotics, dairy products remain the most popular form for probiotic consumption [4].

Beneficial traits of probiotic bacteria are vast. Some of those reported include vitamin production [5,6], cholesterol lowering [7,8,9], alleviation of lactose intolerance [10], cancer prevention [11,12], stimulation of the immune system [13,14], enhancement of bowel motility [15,16], relief from constipation [17,18], prevention and reduction of rotavirus and antibiotic associated diarrhea [19]. Despite the abundant health claims, supported with data from research, still, no favorable opinion has been issued from the European Food Safety Authority (EFSA).

Nowadays, there has been an increasing interest in non-dairy probiotic products, due to the growing number of vegans and to the higher number of individuals with diet restrictions resulting from lactose intolerance, allergies to milk proteins and high cholesterol levels [20,21]. Given all this, there is a need to extend the number of food products in order to cater for those individuals that are unable to consume dairy products or for those that just do not have a liking for dairy derivatives. As a result, the development of novel new probiotic carriers may have a fundamental role in promoting human health.

Since probiotic viability is crucial to confer a health benefit (recommended daily dosage of ~10^9^ viable cells), efforts are being made to improve or sustain their viability during food processing and storage of food products until their consumption [4,22]. Probiotics may be carried within edible polymer matrices used by the food packaging industry. This way probiotics, as well as many other compounds, can be incorporated into biopolymeric matrices to develop active/bioactive food packaging materials as an alternative method to control pathogenic microorganisms [23]. This improves food stability and safety and most importantly favors consumers’ health.

Recently, probiotic incorporation in edible films or coatings has become relatively popular amongst researchers and, consequently, this area has been studied thoroughly in the last years. This review article gives an overview on this subject. In more detail, this review highlights the nature of probiotics, summarizes scientific studies dealing with their incorporation in edible films and coatings, and discusses their application in certain food products and the legislation regarding their use.

## 2. Active and Bioactive Packaging

Industries producing packaging material are common worldwide. Each company comprises of a wide range of sectors with each having its own impact on the market. In order to supply (due to the increasing interest in the consumption of fresh food products of high quality that are characterized with an extended shelf life), packaging industries are utilizing a more modern and safer mode of packaging. Modernization of packaging systems presents a challenge for the food packaging industry, also serving as a driving force for the development of new and improved packaging technology [24].

Active packaging is a type of packaging in which the packaging material, the food product and the environment interact. When food is stored in this type of packaging system, chemical, physical and biological activities aggressively alter its state leading to an increase in shelf life, without compromising food quality. Some of the most beneficial functions and properties of active packaging include control of the moisture content, oxygen diffusion into packages, carbon dioxide and ethylene diffusion, absorption of oxygen and enhancement of flavors and antimicrobial agents. The most commonly used active packaging systems are for antioxidant and antimicrobial purposes, although other purposes may also be accomplished.

Bioactive food packaging systems may provide health benefits to consumers [25]. It is a novel approach in the concept of functional foods, which proposes that any food that can provide a health benefit beyond the traditional nutrients it contains, may be considered as functional. The main difference between the commonly used active packaging technologies and bioactive packaging is that while the former packaging technology deals with maintaining or increasing quality and safety of packaged foods, the latter has a direct impact on the health of the consumer by generating healthier packaged foods. Bioactive packaging materials will ensure the storage and eventually release the bioactive agents into the food product [25].

Since most food products reach the consumer enclosed within or coated with some sort of packaging, packaging has become an important stage in the food chain. One crucial role of food packaging is to maintain or ameliorate food quality and safety. On the contrary, traditional packaging materials are neither readily recyclable nor environmentally sustainable and impose a number of health risks and hazards such as those associated with the migration of harmful additives [23]. As a result, it is essential to continue developing biodegradable (including edible), and sustainable matrices promoting safe and long shelf life integration of bioactive substances. Biodegradable and sustainable materials, such as synthetic biodegradable polymers, natural proteins and derivatives and polysaccharides and derivatives, are considered to be amongst the most efficient materials to be used for the controlled release of substances. Active and bioactive packaging technology have been reviewed in detail elsewhere [23,24,25,26,27].

## 3. Probiotics in Bioactive Edible Films and Coatings

Improving functional products, vehicles of probiotics may have a fundamental role in promoting human health [28]. In order to confer a health benefit, a generally recommended dose of probiotic viable cells is that of 10^8–9^ per day. In fact, issues are raised regarding the cell viability and probiotic survival in the final products. Several factors, both intrinsic and extrinsic, can affect the behavior of a microorganism within different food environments. Typical examples include the type of the selected culture, the physiological state of the cells, the food matrix, the pH, the temperature, the manufacturing processes and the storage conditions [29]. Apart from these factors, the viability of probiotics is determined through their passage of the gastrointestinal tract, where the major hurdles are the low pH and bile. To address these limitations and enhance viability, different technological strategies have been suggested and investigated, with the most important being the inclusion of probiotics into edible films and coatings or their microencapsulation into polymeric matrices [4,28].

As mentioned previously, bioactive food packaging systems may contribute to health benefits to the consumers. The bioactive packaging materials may contain (bioactive) agents that are eventually released into the food product [25]. In the particular case of edible coatings containing probiotics, the release is not even required, since the coating itself is supposed to be eaten with the food [28]. The first study dealing with the incorporation of probiotics into edible films and coatings was that by Tapia et al. in 2007 [30]. Since then, quite a number of studies were available in the literature, investigating the probiotic incorporation in edible films with different approaches. The application of probiotic edible films in food matrices is a tool for effective probiotic delivery to the consumers, but it is also important for enhancing food stability and food safety by controlling the growth of spoilage microorganisms through competition or through antimicrobial substances produced by probiotics.

## 4. Materials for Edible Films and Coatings for Probiotic Applications

Edible films can be produced from materials with film-forming ability. The components used for the preparation of edible films and coatings can be classified into three categories of hydrocolloids, lipids and composites [31,32]. Other compounds such as plasticizers and emulsifiers are added to the film-forming solutions to improve their mechanical properties or to enhance stability when lipids and hydrocolloids are combined [33]. Plasticizers are usually required for the manufacture of edible films and coatings, in particular when polysaccharides or proteins are used as materials. Plasticizers are low molecular weight agents incorporated into film-forming materials that decrease the glass transition temperature. The addition of plasticizers enhances flexibility, toughness and the tear resistance of the film. Amongst the most commonly used plasticizers are glycerol, sorbitol, sucrose and polyethylene glycol [34].

The hydrocolloids group is comprised of polysaccharides and proteins. Polysaccharides used for edible films or coatings are cellulose derivatives, dextrans, inulin, alginate, carrageenan, starch derivatives, pectin derivatives, chitosan, seaweed extracts, and galactomannans [31,33,35,36]. Polysaccharide films and coatings serve as good oxygen, odor and oil barriers with good mechanical properties, but their major drawback is moisture permeability, due to their hydrophilic characteristics [34]. Proteins used for edible films and coatings are gelatin, corn zein, wheat gluten, soy protein, collagen and casein. Protein films are generally formed from solutions or dispersions of the protein as the solvent evaporates. The solvent usually is water, ethanol or their mixture. Proteins must be denatured by heat, acid, base or solvent in order to form a more extended structure that is required for the film formation [31]. Protein-based films exhibit poor water resistance, but they possess better mechanical and barrier properties than polysaccharides [35]. From this group of materials, the most important regarding their use are presented below:

i Cellulose is the major cell wall component in plants. Cellulose derivatives such as methylcellulose (MC) and hydroxypropyl methylcellulose (HPMC) are commonly found in the formulation of edible coatings. Films and coatings based on cellulose and its derivatives are transparent, flexible, odourless and tasteless. MC is more resistant to water, however, the water vapor permeability is quite high. Both MC and HPMC have the ability to form gelatinous coatings after thermal processes, which give the opportunity to be used to prevent oil absorption in frying foodstuff [31]. Regarding their use as probiotic carriers in edible films and coatings, several studies are available [37,38,39,40].
ii Chitosan is mainly obtained from the exoskeleton of crustaceans and fungal cell walls. Chitosan is the *N*-deacetylated derivative of chitin [41]. Chitosan degree of deacetylation, has been reported as an important parameter that determines many of its physicochemical and biological properties, such as crystallinity, hydrophobicity and degradation. The degree of deacetylation of chitosan is controlled by a relatively aggressive alkaline hydrolysis process applied to chitin, with a combination of exposure time and temperature [42,43]. The molecular weight of chitosan is depended on the initial source material (shrimp, crab, fungi) and can be decreased with processing to increase the deacetylation. Molecular weight has been proven to be an important factor in chitosan properties such as crystallinity, degradation, tensile strength and moisture content [43]. Mechanical and barrier properties of chitosan films can be controlled by selecting a suitable solvent system, the appropriate molecular weight of chitosan as well as the addition of plasticizers [44,45]. Chitosan as a coating material has excellent film-forming abilities, broad antimicrobial activity and compatibility with other substances such as vitamins, minerals and antimicrobial agents. This material receives particular interest for the targeted release of probiotics because of its high compatibility with living cells [46]. Furthermore, chitosan has been studied for application as a coating due to its antifungal and antibacterial abilities [47,48,49]. Chitosan can form semi-permeable coatings, which can modify the internal atmosphere when applied to fruits and vegetables. The main disadvantage of chitosan films is their low moisture barrier, which makes their broad use in food applications difficult [35].
iii One of the most commonly used polysaccharides is alginate. Alginate is a generic term for the salts of alginic acid. Alginates possess good film-forming properties and produce transparent and water-soluble films [50]. It has been used mainly for meat products, since it delays dehydration and eliminates lipid oxidation [35,51].
iv Starch and its derivatives have been widely used as food hydrocolloids, because they are inexpensive, abundant, biodegradable and easy to use. Coatings made from starch are usually transparent, odourless, tasteless and colourless with low permeability to oxygen at low-to-intermediate relative humidity [52]. Starch films have excellent barrier properties to O_2_ and CO_2_, but not to water. Starch granules contain the macromolecules amylose and amylopectin, which can form solutions and gels. Amylose is a sparsely branched molecule mainly based on α(1–4) bonds with a molecular weight of 10^5^–10^6^ anhydroglucose units [53]. During gel formation amylose and amylopectin form inter- and intra-molecular crosslinks so that they produce a macromolecular network, and subsequently the film after water evaporation. Physical crosslinks in the macromolecular network of starch are formed mainly by microcrystalline domains of amylose. The more these domains exist in the starch-based film, the higher the tensile strength of the films [54]. A high amylose content in starch is responsible for the production of strong and flexible films [55]. Chemical, physical and functional properties of edible films and coatings depend on the amylose/amylopectin ratio [53]. Degree of crystallinity of starch films increases by increasing the amylose content of starch [54]. Starches with high-amylose content have been used to extend the shelf life of deep fried foods [56]. Additionally to the usual starch films, modified starch films have started to be investigated because they possess good solubility and improved mechanical properties. Starches such as cross-linked, substituted, oxidized and acid-hydrolyzed are being produced as a result of chemical modifications. These chemical modifications have been examined regarding their effect on the film characteristics such as their mechanical, barrier and thermal properties. More specifically, starches with increased numbers of cross-linkages exhibit improved water absorption and maintain viscosity and texture. Substitution results in increased water affinity, lower starch gelatinization temperature, better hydration, and less firm gels. Oxidized starches are applied to deep fried food as coatings to improve their eating quality by retaining crispiness. These starches usually are corn, potato, cassava and bean starches. Acid-modified starches, are usually applied to jelly candies, processed meats and to extruded cereals and snacks. Acid hydrolysis leads to decreased swelling power, increased solubility and more options regarding the gelatinization temperature compared to native starches [56].
v Pectins are structural components of plant cell walls. These materials are a common type of gelling agents, as they are widely used in jams, jellies and sweets, apart from the production of edible films. Several studies have investigated the potential of pectin as a material in edible films [57,58].
vi Gelatin is obtained by controlled hydrolysis of collagen at high temperatures in the presence of water and it is widely found in nature. Antioxidant and antimicrobial activities are associated with gelatin [59]. In general, gelatin films and coatings have poor water vapor barrier properties [35] and their application includes meats, since they reduce oxygen, oil and moisture transport [31]. López de Lacey et al. [60] and Soukoulis et al. [61], have investigated the properties of gelatin edible films incorporated with probiotics.

Lipids used for edible films and coatings include natural waxes, vegetable oils, acetoglycerides and fatty acids and resins. These compounds exhibit certain disadvantages regarding their application, showing mechanical and chemical instabilities as well as decreased organoleptic quality [34]. Lipids are usually combined with other film-forming materials such as polysaccharides or proteins, to increase the resistance to water penetration [35].

Edible films and coatings might be heterogeneous regarding the material used for their manufacture. It is possible that an edible film consists of a blend of polysaccharides, protein and/or lipids. Such a film is defined as a composite [31]. The aim of producing composite films is to adjust the characteristics of the film in order to use it for a specific application. Since each individual coating material possesses some unique, but limited functions, a combination of different materials can be more effective [32]. Studies regarding probiotic edible films and coatings and their materials are presented in Table 1.

## 5. Microencapsulation Techniques

Microencapsulation is defined as a technology including ingredients (solid, liquid or gaseous) that are being entrapped or completely surrounded by protective matrices [4]. Referring to probiotics, the aim of encapsulation techniques is to carry and protect these bacteria from the effects of the low pH and bile, which these bacteria encounter during their passage through the gastrointestinal tract [4,71]. Additionally, microencapsulation techniques intend to protect and reassure the viability of the encapsulated probiotics during food processing and storage conditions of the food products until their consumption. Other factors that are involved in the microencapsulation procedure such as low water activity or oxygen exposure can affect the efficacy of this technique. With regards to the materials used for microencapsulation, there are numerous studies available with a major category being biodegradable polymers. These biopolymers, include alginate, chitosan, cellulose, protein, carrageenan, gelatin and pectin which are materials for the manufacturing of edible films and coatings [4,72]. Applications of microencapsulation and the materials used are presented in Table 2. Microencapsulation techniques and application have been reviewed elsewhere [4,22,72,73,74,75].

## 6. Effects of Edible Films on Probiotic Viability

Regardless of the technological mechanism for the production of edible films containing probiotics, it is of primary importance that the film has the ability to provide viable probiotic bacteria to the gastrointestinal (GI) tract. The viability, stability and survival of several probiotic strains under various conditions have been extensively studied and reviewed, while only few studies are available dealing with the evaluation of the viability of probiotic strains specifically in films in order to assess their suitability as probiotic carriers [28]. The chemistry of the film, as well as the film-forming process, is critical as it is directly associated with bacterial survival post-processing and post-ingestion [57]. Some of these studies will be mentioned in this section.

Soukoulis et al. [57] investigated the stability of the probiotic *Lactobacillus rhamnosus* GG, incorporated in films of different biopolymers with the addition of whey protein concentrate. The population of the strain one hour after the inoculation was high, showing no acute toxic effects of the biopolymer type or of the whey protein concentrate on its cell viability, whereas the compositional characteristics of the film-forming solution were found to be influential. In the same study, it was found that polysaccharide-based films from pectin, low and high viscosity sodium alginate and films from locust bean gum and κ-carrageenan had the highest cell lethality compared to the one including whey protein concentrate. This might be explained because of the low pH of the pectin film solution without whey protein concentrate (pH 3.9–4.2), when the alginate solution had a pH of 5.4–5.7 and the locust bean gum/κ-carrageenan had higher values (6.3–6.7). Other intrinsic parameters such as the low redox potential and the surface tension of the substrate may affect the viability of *L. rhamnosus* GG [57]. Furthermore, the same researchers concluded that when the biopolymer entanglement takes place via the physical entrapment of probiotic cells and preservation of water in hydrogel interspaces may enhance *L. rhamnosus* GG cells to maintain their physical cell structure and this might be the explanation of why biopolymers with good hydrogel-forming ability possess better performance.

In another study of Kanmani et al. [65], pullulan and starch-based edible films with probiotic supplemented bacteria were studied. *Lactobacillus rhamnosus* GG, *Lactobacillus reuteri* and *Lactobacillus acidophilus* were incorporated into pullulan, starches and their combination (pullulan/starches) films and were stored at 25 and 4 °C. At room temperature, all of the films showed similar cell viabilities, whereas the maximum viability was observed in pure pullulan films. However, all of the films, except of starch, maintained cell viabilities up to 20 days. The addition of starch in the pullulan films was found to negatively affect cell survival. Among the different origins of starch, potato starch was found to confer better cell survival compared to the other starches. This might be explained due to the higher moisture content and moisture absorption capability of potato starch. After extended storage time (30–60 days), no viable cells were detected, probably due to increased bacterial metabolism. On the contrary, at 4 °C the probiotic cells were more stable during storage up to 20 days. The pure pullulan film and the pullulan/potato starch film retained cell viability higher than 80% even after 30 days of refrigerated storage, probably due to decreased bacterial metabolism.

Corn and rice starch-based films, containing substances of protein and cells of *Lactobacillus rhamnosus* GG were studied by Soukoulis et al. [61]. In this work, the addition of sodium caseinate enhanced the probiotic viability, compared to gelatin and soy protein concentrate. In corn starch films, the protein type had a significant effect on probiotic viability at room temperature, and was starch- and temperature-dependent. The same effect was not observed in rice starch films, when proteins acted independently of storage temperature on probiotic viability [61].

The viability of *Lactobacillus casei* added to sodium caseinate films via spraying or direct incorporation was tested by Gialamas et al. [62]. In this study, sorbitol was used as plasticizer in the film-forming solution and two temperatures, 4 and 25 °C were chosen for storage. Direct incorporation of bacterial cells into the film-forming solution caused increased viability in both temperatures. For enhanced viability, sorbitol was added in the films, since this or similar compounds are found to act as protective agents for microbial cells during drying or low water activity storage. This hypothesis was confirmed from the results, and the viability of the cells was increased during the storage at 25 °C for a period of 30 days.

The viability of *Bifidobacterium animalis* Bb-12 and *Lactobacillus casei*-01 in WPI films was investigated recently [69]. As it was expected, the viable cell numbers of both probiotics were decreased at higher rates when the films were stored at 23 °C. From an initial concentration of 10^9^ CFU/g film, a loss in viability of approximately three logs was observed within ten days of storage in the aforementioned temperature for both probiotics, and was maintained steadily thereafter, with a slight decrease upon 40 days. At 4 °C, the loss of viability was limited with 1 and 2 log reductions for *B. animalis* and *L. casei*, respectively, at the end of the storage period, while their viability remained constant for ten days, in the same temperature. These results suggest that the stability of probiotics increases in low storage temperatures. Furthermore, whey protein has positive effects on the survival of probiotic bacteria during storage, through its nutritional value, by reducing the redox potential of the medium and increasing the buffering capacity, which results in a smaller decrease in pH [69].

It has to be noted that in the aforementioned studies, cell viability was investigated during the storage of the edible films without their application in foodstuff. However, the scheme changes regarding their viability after the contact with the food product. In a study of Concha-Meyer et al. [63], lactic acid bacteria were incorporated into alginate films and used to wrap salmon inoculated with *Listeria monocytogenes*. It was observed that the lactic acid bacteria strain viability and growth within the film matrix was improved after contact with the salmon, probably because of nutrient diffusion. Furthermore, the water activity of the film after drying (a_w_ = 0.91) also contributed to maintaining cell viability, since a slight increase in this value was observed at the time of contact with the salmon (a_w_ = 0.92). Additionally, the pH of the film increased after the salmon was wrapped with it for 24 h at 4 °C from 4.4 to 5.6, due to the diffusion and buffering capacity of the salmon juice [63]. In a recent study, Pavli et al. [70] investigated the viability of different probiotic strains of *Lactobacillus plantarum* and *Lactobacillus pentosus* incorporated into sodium alginate edible films. The viability of the incorporated strains was examined throughout the storage period and in contact with ham slices (66 days at 4 °C, 47 days at 8 °C and 40 days at 12 °C). The storage temperature had no effect on the population levels of the inoculated strains, however, it was strain-dependent. In general, a reduction in the population of probiotics was detected in the films, in the sampling point after their application on ham for all temperatures, probably due to the drying process and the subsequent stress. However, this had a limited effect on the probiotic survival in adequate levels (>10^6^ CFU/g).

## 7. Prebiotics to Enhance Probiotic Viability

A prebiotic was first defined as “a non-digestible food ingredient that beneficially affects the host by selectively stimulating the growth and/or activity of one or a limited number of bacteria in the colon, and thus improves host health” [102]. The products that contain both probiotics and prebiotics are defined as “synbiotics”, and because the word alludes to synergism, this term should be reserved for products in which the prebiotic compound selectively favors the probiotic compound [103]. It is known that the synergistic combination of prebiotics with probiotic strains promotes colonization in the intestinal tract as well as preventing several forms of cancer. The chemical structure of some oligosaccharides makes them resistant to digestive enzymes and thus, they are able to reach the large intestine where they become available for fermentation by saccharolytic bacteria [104]. Addition of prebiotic components is a promising technology for an effective probiotic protection. There are only a few studies dealing with the application of prebiotics specifically in probiotic edible films, as co-components in order to possess stability and functionality to the entrapped probiotic bacteria [29,39].

Soukoulis et al. [29] investigated the viability of the probiotic *Lactobacillus rhamnosus* GG in gelatin films with several prebiotics as co-components: inulin, polydextrose, wheat dextrin and glucose-oligosaccharides. Regarding the film-forming process and the subsequent drying, the addition of glucose-oligosaccharides and polydextrose in the films provided better survival rates of the probiotic strain, while inulin and wheat dextrin had an adverse effect on cell viability. The inactivation rates of *L. rhamnosus* GG were higher in the films stored at room temperature (25 °C), compared to the films stored under refrigeration. Except for the polydextrose edible films stored at 25 °C, the presence of prebiotics in the matrices improved storage stability of *L. rhamnosus* GG. Inulin was the most effective fiber followed by wheat dextrin, glucose-oligosaccharides and polydextrose, at both temperatures. Furthermore, the shelf life of the edible films, regarding the probiotic survival, ranged from 63 to 100 days and 17 to 30 days at 4 and 25 °C, with the inulin ones having the longest shelf life [29].

Romano et al. [39], developed MC edible films with the incorporation of two different strains of lactic acid bacteria (*Lactobacillus delbrueckii* subsp. *bulgaricus* CIDCA 333 and *Lactobacillus plantarum* CIDCA 83114) and fructo-oligosaccharides as prebiotics in various concentrations in the film-forming solution. The drying step led to a significant decrease in the viability of *L. delbrueckii* subsp. *bulgaricus* CIDCA 333 in case of the fructo-oligosaccharides absence from the films. Increasing the concentration of the fructo-oligosaccharides in films had a strong protective effect up to a 3% *w*/*v* concentration of the prebiotic; when in higher concentrations no further protected effect was observed. *L. plantarum* CIDCA 83114, showed a greater intrinsic resistance towards the drying process and the addition of fructo-olifosaccharides up to 5% *w*/*v* did not significantly increase its viability.

## 8. Sensory Assessment

It is well known that the addition of probiotic bacteria in free form in food products, can strongly modify their sensory characteristics. Phenomena during metabolic activity, such as extreme acidification or excessive proteolysis can decrease the product acceptability by consumers [105]. Incorporation of probiotics in edible films or coatings can help to eliminate or control undesirable modifications associated with their addition in free form [105], although the probiotic effect on the sensory attributes is still present.

Due to the fact that edible films and coatings are usually consumed with the coated products, they must have organoleptic properties that are as neutral as possible, in order not to be detected when eaten [106]. Although there are some studies dealing with the sensory characteristics of edible films containing antimicrobial substances, such as essential oils, there is a gap of knowledge regarding the sensory characteristics of edible films containing probiotic bacteria. More specifically, there are only a few studies with sensorial analyses of products packed with probiotic edible films and coatings. However, the possible effects of probiotic edible films on the sensory attributes of the products can be easily assumed.

In a study of Tavera-Quiroz et al. [40], edible coatings of methylcellulose containing a probiotic *Lactobacillus plantarum*, were applied to green apple baked snacks. Sensory analysis was performed in the control (without the probiotic coating) and in samples with the probiotic coating. The analyzed attributes were the appearance, color, taste, texture and overall acceptability, using a nine-point hedonic scale, ranging from 1 (dislike extremely) to 9 (like extremely). The taste score of the snack coated with the methylcellulose film containing the probiotic was significantly lower than the control taste values (6.5 and 7.2, respectively). With regards to color, results were almost similar, although the texture values of the control snack were slightly higher than the ones of the probiotic coating, probably due to the higher moisture content of the latter.

García-Argueta et al. [107] studied the effect of whey, inulin and gelatin-based edible coatings containing the probiotic *Lactobacillus casei* Shirota, on a cracker cookie. From the sensory assessment, it was found that the newly produced cookie had no significant differences compared to the control and was highly accepted from the panelists. Regarding the appearance, only 34.6% reported that cookies with the probiotic coating are different to the controls, which is a very important outcome for a consumers’ study. Furthermore, 48% reported little or no difference in the appearance or aroma, 61% little or no difference in flavor and 52% little or no difference in texture. In terms of product acceptance, 62% reported moderately to greatly with regards to liking the cracker cookie, whereas only 4% mentioned disliking it.

In a recent study [70], the sensorial impact of probiotic-supplemented sodium alginate films on ham slices was reported. The presence of the probiotic-supplemented film affected significantly the aroma and taste parameters of the ham slices as well as the total organoleptic scores, regardless of the storage temperature tested (4, 8 and 12 °C). The taste of the samples with the probiotic films was found to be more acidic than the control ones, while the aroma was more acidic as well. These phenomena were slightly eliminated when the probiotic edible films were applied in ham slices after high pressure processing treatment, probably due to a lower level of bacterial population in the ham samples [70].

In order for a probiotic product to achieve a commercial success on the market, it is obvious that, it has to meet the sensory needs of the consumers. Consequently, the sensory evaluation is a mandatory assessment tool before launching a new product. The product type, the particular characteristics of the incorporated probiotic strain, the material of the edible film or coating as well as the storage conditions, need to be taken into account.

## 9. Regulatory Issues

Before the commercial production of a food product that contains an edible film or coating, the manufacturing company should take into account the regulatory status and the current legislation [27]. To maintain edibility, all film-forming components as well as any additives should be food-grade, safe materials and all processing facilities should meet high hygienic levels [32]. The edible film/coating must meet the safety requirements for food contact materials, must be labeled as a food additive or labeled even as an active packaging material [108,109,110]. Another important issue is the possible presence of allergens in the films or coatings. There are many ingredients which are used in the production of these materials such as milk proteins (whey/casein), wheat protein, soy protein, thus, their presence in a food product should be clearly labeled [32]. Moreover, edible films/coatings based on certain biodegradable biopolymers are environmental friendly, due to the reduction in the amounts of environmental toxic materials that are used by the packaging industry [58].

When edible films and coatings contain probiotics, more issues are raised regarding the legislation. In the European Union (EU), there is no legal framework defining probiotic bacteria or the food category “probiotics” and the legislation is quite conservative. Even if most of the probiotic microorganisms were used as safe food components and their safety is not negotiated, a favorable opinion from EFSA is required for an authorization by the European Commission, according to EC No. 1924/2006 regulation [111]. However, the situation is different in the United States, where the legislation for the use of probiotics varies, regarding the scope of their use (drug or dietary supplement). In general, for EU, all the ingredients that will be used in foods for human consumption and the subsequent health or nutritional claims, will need to be harmonized with the regulations (EC 1924/2006, EC 432/2012, EC 2283/2015), which involve scientific requirements [111,112,113]. Therefore, successful market applications in the future will be a result of the combined academic and industrial focus, to confront with the numerous regulatory, scientific and economic issues [28,114].

## 10. Future Trends

The probiotic incorporation into edible films and coatings is an emerging technology that has been very popular recently, given that an increasing number of studies are available. The future presence of probiotic edible films and coatings applied to food products depends on several elements. Firstly, the legislation frame regarding probiotic foods should allow and encourage the manufacturers to take the challenge and invest in the new technologies for a more effective probiotic food production. A better spread of the scientific results and knowledge towards industry will provide new chances of businesses to develop the production of novel and functional foods. It is necessary to optimize several technological and economic aspects of the manufacturing processes and this can only be achieved through the increased demand from consumers and, therefore, the market. New studies must be carried out in many aspects of edible film technology such as testing of the existing or other media that have not yet been industrially utilized, selecting suitable probiotic bacteria to be incorporated in each type of edible film, assessing the impact of the different types of films into different food products, and favoring the needs of particular groups of consumers such as vegetarians, vegans and lactose-intolerants. It is very important to investigate in more detail, the properties of the existing materials used to produce edible films in order to adequately protect the incorporated probiotic bacteria. Moreover, the combination of probiotics and prebiotics in edible films and coatings will be an interesting area to research, given that only a limited number of studies are available to the best of our knowledge. On the other hand, in vivo studies should be carried out to evaluate the viability of the incorporated probiotics under gastrointestinal conditions as well as to establish the level of probiotics to be delivered throughout these systems, in order to confer health benefits.

## Figures and Tables

**Table 1:** Edible films and coatings that have been used for probiotic or lactic acid bacteria incorporation.

Biopolymer Material	Probiotics	Additives	Application Matrix	Reference
Alginate/Gellan	*B. lactis* Bb-12	Glycerol	Fresh-cut apples and papayas	[30]
Sodium caseinate	*L. sakei*	Sorbitol	Fresh beef	[62]
Alginate/Starch	*C. maltaromaticum*	Glycerol	Smoked salmon	[63]
Gelatin	*L. acidophilus*, *B. bifidum*	Glucose, cysteine, sorbitol, glycerol	Hake fish	[60]
Starch	*L. acidophilus*	-	Baked bread	[64]
Pullulan/Starch	*L. plantarum*, *L. reuteri*, *L. acidophilus*	-	-	[65]
Isolate Pea Protein/MC/Sodium caseinate/HPMC	*L. plantarum*	Glycerol	-	[37]
Agar	*B. animalis* spp. *lactis*, *L. paracasei* spp. *paracasei*	Green tea extract, glycerol	Hake fillets	[66]
Methylcellulose (MC)	*L. delbruecki* subsp. *bulgaricus*, *L. plantarum*	Fructooligosaccharides (FOS), sorbitol	-	[39]
Gelatin	*L. rhamnosus* GG	Inulin, polydextrose, wheat dextrin, glucose-oligosaccharides, glycerol	-	[29]
Alginate, Whey Protein Concentrate	*L. rhamnosus* GG	Glycerol	Bread	[67]
MC, Sodium caseinate	*L. reuteri*, *L. acidophilus*	Glycerol	-	[38]
Kefiran	*L. plantarum*	Glycerol	-	[68]
WPI	*B. animalis* Bb-12, *L. casei*	Glycerol	-	[69]
Rice/Corn Starch, Gelatine/Sodium caseinate/Soy protein concentrate	*L. rhamnosus* GG	Glycerol	-	[61]
Sodium alginate	*L. plantarum*, *L. pentosus*	Glycerol	Ham slices	[70]
Sodium alginate/Pectin/κ-Carrageenan-Locust bean gum/Gelatine/Whey protein concentrate	*L. rhamnosus* GG	Glycerol	-	[57]

MC = Methylcellulose, HPMC = Hydroxypropyl methylcellulose, WPI = Whey Protein Isolate.

**Table 2:** Microencapsulation applications for probiotic delivery.

Biopolymer Material	Probiotics	Additives	Application Matrix	Reference
Alginate	*L. acidophilus*, *L.casei*, *B. bifidum*	-	-	[76]
Chitosan/Alginate	*L. casei* Shirota	-	-	[77]
Alginate/maize starch	*L. acidophilus*, *B. lactis*	-	Yoghurt	[78]
Alginate/Whey protein	*L. plantarum*	-	-	[79]
Alginate	*B. bifidum*, *L. acidophilus*	-	White-brined cheese	[80]
Whey protein/Alginate	*S. boulardii*	-	-	[81]
Alginate/Chitosan	*L. gasseri*, *B. bifidum*	-	-	[82]
WPI	*L. rhamnosus* GG	-	-	[83]
Sodium alginate/Maize starch	*L. acidophilus*	-	White-brined cheese	[84]
Alginate/Chitosan	*L. plantarum*	-	Pomegranate juice	[85]
Alginate/Chitosan	*L. plantarum*	-	-	[86]
Alginate/Pectins	*L. plantarum*, *B. longum*	-	Pomegranate and cranberry juice	[87]
Alginate	*L. casei*	Maize starch, poly-l-lysine, stearic acid, bees wax	-	[88]
Cellulose acetate phthalate	*B. animalis* BB-12	-	Acerola nectar	[89]
Alginate/Chitosan	*L. reuteri*	-	Chocolate souffle	[90]
Pectin/Whey protein concentrate	*L. acidophilus*	-	Yoghurt	[91]
Sodium alginate	*L. plantarum*	Inulin	-	[92]
Sweet whey	*Bifidobacterium* BB-12	Inulin, polydextrose		[71]
Sodium alginate/Chitosan	*B. longum*	-	-	[93]
Sodium alginate	*L. acidophilus*	Hi-maize	-	[94]
Calcium pectinate	*L. helveticus*	Green tea extracts	-	[95]
WPI/Hydrolysed whey protein	*L. rhamnosus* GG	-	-	[96]
Sodium alginate/Citric pectin	*L. plantaru*	-	-	[97]
Whey protein concentrate	*L. plantarum*	-	-	[98]
CMC/κ-carrageenan	*L. plantarum*	-	-	[99]
Sodium alginate	*B. animalis* BB-12	-	-	[100]
WPI/GA	*Lactobacillus paracasei* subsp. *paracasei/Lactobacillus paraplantarum*	-	-	[101]

WPI = Whey Protein Isolate, CMC = Carboxymethyl cellulose, GA = Gum Arabic.

## Abbreviations

FAO	Food and Agriculture Organization of the United Nations
WHO	World Health Organization
MC	Methylcellulose
HPMC	Hydroxypropyl methylcellulose
CMC	Carboxymethyl cellulose
GI	Gastrointestinal
WPI	Whey Protein Isolate
GA	Gum Arabic
EFSA	European Food Safety Authority
EC	European Commission
EU	European Union
